# Crystal structure of 1,5-diethyl-3′,5′-di­phenyl-1,5-di­hydro-3′*H*-spiro­[pyra­zolo[3,4-*d*]pyrimidine-4,2′-[1,3,4]thia­diazole]

**DOI:** 10.1107/S2056989015017405

**Published:** 2015-09-26

**Authors:** Mohammed El Fal, Youssef Ramli, El Mokhtar Essassi, Mohamed Saadi, Lahcen El Ammari

**Affiliations:** a Laboratoire de Chimie Organique Hétérocyclique URAC 21, Pôle de Compétence Pharmacochimie, Av. Ibn Battouta, BP 1014, Faculté des Sciences, Université Mohammed V, Rabat, Morocco; b Laboratory of Medicinal Chemistry, Faculty of Medicine and Pharmacy, University Mohammed V, Rabat, Morocco; c Laboratoire de Chimie du Solide Appliquée, Faculté des Sciences, Université Mohammed V, Avenue Ibn Battouta, BP 1014, Rabat, Morocco

**Keywords:** crystal structure, pyrazolo­[3,4-*d*]pyrimidine, thia­diazole

## Abstract

In the title compound, C_22_H_22_N_6_S, the pyrazolo­[3,4-*d*]pyrimidine rings system is almost planar, with the r.m.s. deviation for the fitted atoms being 0.011 Å. The two phenyl groups linked to the thia­diazole ring are nearly perpendicular to the fused-ring system as indicated by the dihedral angles of 86.93 (10) and 83.35 (11)°. However, the phenyl rings are almost coplanar with the thia­diazole ring (r.m.s. deviation = 0.015 Å), forming dihedral angles of 10.44 (11) and 10.06 (12)°. In the crystal, mol­ecules are connected into a supra­molecular layer in the *ac* plane *via* C—H⋯π inter­actions.

## Related literature   

For biological properties of pyrazolo­[3,4-*d*]pyrimidine derivatives, see: Chern *et al.* (2004 ▸); Schenone *et al.* (2009 ▸); Dinér *et al.* (2012 ▸); Taliani *et al.* (2010 ▸); Trivedi *et al.* (2012 ▸). For related structures, see: El Fal *et al.* (2014 ▸, 2015 ▸); Ahoya *et al.* (2011 ▸); Anothane *et al.* (2012 ▸).
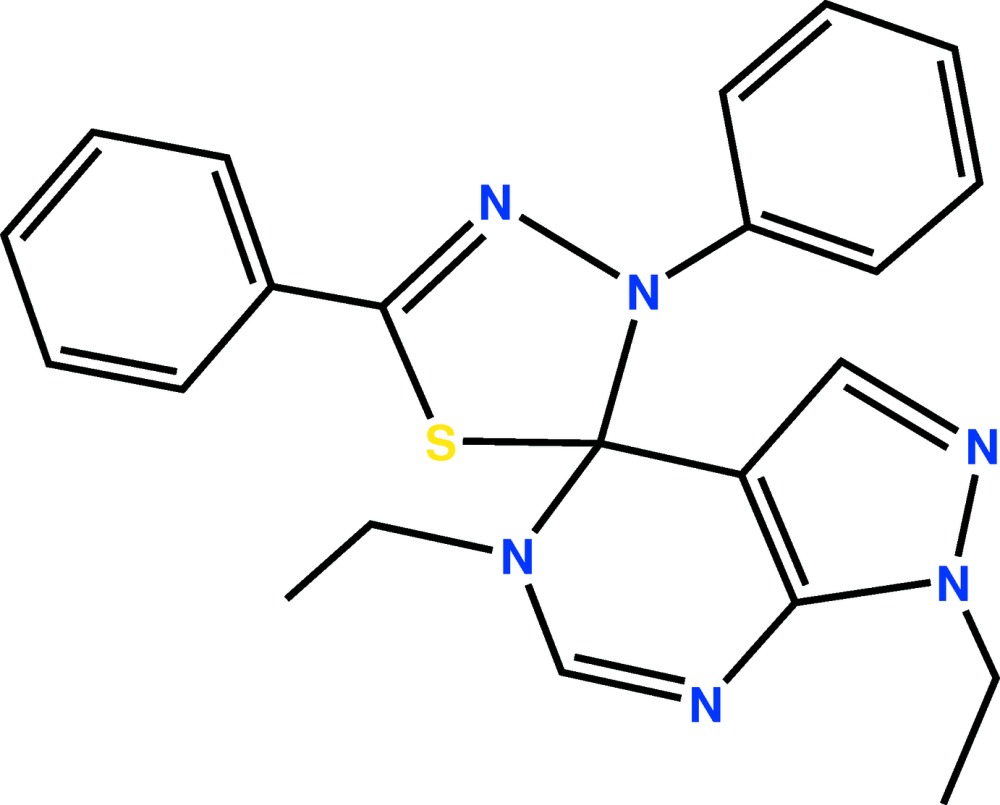


## Experimental   

### Crystal data  

C_22_H_22_N_6_S
*M* *_r_* = 402.51
Orthorhombic, 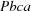

*a* = 14.501 (5) Å
*b* = 22.898 (5) Å
*c* = 12.468 (4) Å
*V* = 4140 (2) Å^3^
*Z* = 8
Mo *K*α radiation
μ = 0.18 mm^−1^
*T* = 296 K
0.37 × 0.34 × 0.29 mm

### Data collection  

Bruker X8 APEX diffractometer
Absorption correction: multi-scan (*SADABS*; Bruker, 2009 ▸) *T*_min_ = 0.589, *T*_max_ = 0.746
25025 measured reflections
4224 independent reflections
2566 reflections with *I* > 2σ(*I*)
*R*_int_ = 0.079

### Refinement  

*R*[*F* ^2^ > 2σ(*F* ^2^)] = 0.048
*wR*(*F* ^2^) = 0.138
*S* = 1.00
4224 reflections
262 parameters
H-atom parameters constrained
Δρ_max_ = 0.31 e Å^−3^
Δρ_min_ = −0.30 e Å^−3^

### 

Data collection: *APEX2* (Bruker, 2009 ▸); cell refinement: *SAINT-Plus* (Bruker, 2009 ▸); data reduction: *SAINT-Plus*; program(s) used to solve structure: *SHELXS97* (Sheldrick, 2008 ▸); program(s) used to refine structure: *SHELXL2013* (Sheldrick, 2015 ▸); molecular graphics: *ORTEP-3 for Windows* (Farrugia, 2012 ▸); software used to prepare material for publication: *SHELXL2013*.

## Supplementary Material

Crystal structure: contains datablock(s) I. DOI: 10.1107/S2056989015017405/tk5387sup1.cif

Structure factors: contains datablock(s) I. DOI: 10.1107/S2056989015017405/tk5387Isup2.hkl

Supporting information file. DOI: 10.1107/S2056989015017405/tk5387Isup3.cml

**Fig. 1.**
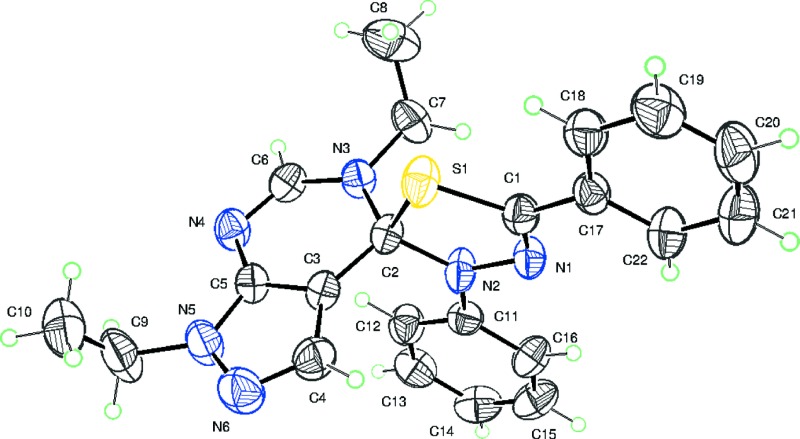
Molecular structure of the title compound with the atom-labelling scheme. Displacement ellipsoids are drawn at the 50% probability level. H atoms are represented as small circles.

. DOI: 10.1107/S2056989015017405/tk5387fig1.tif
Mol­ecular structure of the title compound with the atom-labelling scheme. Displacement ellipsoids are drawn at the 50% probability level. H atoms are represented as small circles.

CCDC reference: 1425539

Additional supporting information:  crystallographic information; 3D view; checkCIF report

## Figures and Tables

**Table 1:** Hydrogen-bond geometry (, ) *Cg*1 and *Cg*2 are the centroids of the C17C22 and C11C16 rings, respectively.

*D*H*A*	*D*H	H*A*	*D* *A*	*D*H*A*
C14H14*Cg*1^i^	0.93	2.75	3.615 (3)	155
C20H20*Cg*2^ii^	0.93	2.77	3.564 (4)	144

Symmetry codes: (i) 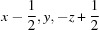; (ii) 
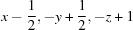
.

## supplementary crystallographic information

### S1. Comment

The pyrazolo[3,4-*d*]pyrimidine nucleus is considered as a very interesting and versatile scaffold for the synthesis of potential drug candidates acting on a wide range of biological targets (Schenone *et al.*, 2009). Among their many applications, they have been used as kinase inhibitors (Dinér *et al.*, 2012), antiviral agents (Chern *et al.*, 2004), adenosine antagonists (Taliani *et al.*, 2010 and as antitubercular agents (Trivedi *et al.*, 2012). In the search for new compounds with therapeutic interest, we have prepared spiro[pyrazolo[3,4-*d*]pyrimidine-1,2'-[1,3,4]thiadiazole] derivatives *via* 1,3-dipolar cycloaddition using diphenyl hydrazonoyl chloride as the precursor for diphenyl nitrilimine, and 1,5-diethyl-1*H*-pyrazolo [3,4-*d*]pyrimidin-4(5*H*)-thione as the dipolarophile (El Fal *et al.*, 2014; El Fal *et al.*, 2015; Ahoya *et al.*, 2011, Anothane *et al.*, 2012).

The molecule of the title compound is built up from two fused five- and six-membered heterocycles linked to two ethyls and to two phenyl rings *via* the thiadiazole ring as shown in Fig. 1. The pyrazolo[3,4-*d*]pyrimidine system is virtually planar with the largest deviation from the mean plane being 0.016 (2) Å at N3 and makes dihedral angles of 86.93 (10) and 83.35 (11)° with the mean plane through the first (C11 to C16) and the second (C17 to C22) phenyl rings, respectively. Furthermore, the two phenyl rings are virtually coplanar with the largest deviation from the mean plane of -0.069 (2) Å, and makes a dihedral angle of 9.88 (9)° with the thiadiazole ring. No classic hydrogen bonds are observed in the structure.

### S2. Experimental

To a solution of 1,5-diethyl-1*H*-pyrazolo [3,4-*d*] pyrimidin-4(5*H*)–thione (2.08 g, 10 mmol) and diphenyl hydrazonoyl chloride (3.00 g, 13 mmol) in THF (30 ml) was added triethylamine (2 ml). The mixture was refluxed for 24 h. The precipitate was collected by filtration and was separated by silica gel chromatography (hexane/ethyl acetate: 8/2). The solid obtained was recrystallized from ethanol to afford the title compound as yellow crystals (yield: 60%; m.p. = 468 K).

### S3. Refinement

The H atoms were located in a difference map and treated as riding with C—H = 0.93 Å (aromatic), C—H = 0.97 Å (methylene) and C—H = 0.96 Å (methyl). All hydrogen with *U*_iso_(H) = 1.2 *U*_eq_(aromatic and methylene) and *U*_iso_(H) = 1.5 *U*_eq_(methyl). The reflection (0 2 0) was affected by beamstop and was removed from the refinement.

### Crystal data

**Table:**

C_22_H_22_N_6_S	*D*_x_ = 1.292 Mg m^−^^3^
*M**_r_* = 402.51	Melting point: 468 K
Orthorhombic, *P**b**c**a*	Mo *K*α radiation, λ = 0.71073 Å
*a* = 14.501 (5) Å	Cell parameters from 4224 reflections
*b* = 22.898 (5) Å	θ = 2.3–26.4°
*c* = 12.468 (4) Å	µ = 0.18 mm^−^^1^
*V* = 4140 (2) Å^3^	*T* = 296 K
*Z* = 8	Block, yellow
*F*(000) = 1696	0.37 × 0.34 × 0.29 mm

### Data collection

**Table:**

Bruker X8 APEX diffractometer	4224 independent reflections
Radiation source: fine-focus sealed tube	2566 reflections with *I* > 2σ(*I*)
Graphite monochromator	*R*_int_ = 0.079
φ and ω scans	θ_max_ = 26.4°, θ_min_ = 2.3°
Absorption correction: multi-scan (*SADABS*; Bruker, 2009)	*h* = −18→17
*T*_min_ = 0.589, *T*_max_ = 0.746	*k* = −24→28
25025 measured reflections	*l* = −15→15

### Refinement

**Table:**

Refinement on *F*^2^	0 restraints
Least-squares matrix: full	Hydrogen site location: inferred from neighbouring sites
*R*[*F*^2^ > 2σ(*F*^2^)] = 0.048	H-atom parameters constrained
*wR*(*F*^2^) = 0.138	*w* = 1/[σ^2^(*F*_o_^2^) + (0.0625*P*)^2^ + 0.732*P*] where *P* = (*F*_o_^2^ + 2*F*_c_^2^)/3
*S* = 1.00	(Δ/σ)_max_ < 0.001
4224 reflections	Δρ_max_ = 0.31 e Å^−^^3^
262 parameters	Δρ_min_ = −0.30 e Å^−^^3^

### Special details

**Table:**

Geometry. All e.s.d.'s (except the e.s.d. in the dihedral angle between two l.s. planes) are estimated using the full covariance matrix. The cell e.s.d.'s are taken into account individually in the estimation of e.s.d.'s in distances, angles and torsion angles; correlations between e.s.d.'s in cell parameters are only used when they are defined by crystal symmetry. An approximate (isotropic) treatment of cell e.s.d.'s is used for estimating e.s.d.'s involving l.s. planes.

### Fractional atomic coordinates and isotropic or equivalent isotropic displacement parameters (Å^2^)

**Table:**

	*x*	*y*	*z*	*U*_iso_*/*U*_eq_	
C1	0.48041 (15)	0.16398 (10)	0.52664 (19)	0.0383 (5)	
C2	0.54607 (15)	0.07863 (9)	0.63901 (19)	0.0363 (5)	
C3	0.49773 (15)	0.04386 (10)	0.72025 (19)	0.0373 (5)	
C4	0.41299 (17)	0.04978 (11)	0.7732 (2)	0.0511 (7)	
H4	0.3727	0.0809	0.7631	0.061*	
C5	0.53365 (15)	−0.00752 (10)	0.75801 (19)	0.0377 (5)	
C6	0.65913 (16)	−0.00200 (10)	0.6605 (2)	0.0413 (6)	
H6	0.7162	−0.0164	0.6392	0.050*	
C7	0.69620 (18)	0.07699 (12)	0.5361 (2)	0.0554 (7)	
H7A	0.7560	0.0814	0.5700	0.066*	
H7B	0.6733	0.1158	0.5199	0.066*	
C8	0.7081 (3)	0.04447 (17)	0.4342 (3)	0.1022 (13)	
H8A	0.7498	0.0654	0.3884	0.153*	
H8B	0.6495	0.0406	0.3992	0.153*	
H8C	0.7327	0.0064	0.4492	0.153*	
C9	0.4750 (2)	−0.08616 (13)	0.8837 (3)	0.0726 (9)	
H9A	0.4554	−0.0814	0.9576	0.087*	
H9B	0.5377	−0.1009	0.8841	0.087*	
C10	0.4139 (3)	−0.12911 (14)	0.8295 (3)	0.0962 (13)	
H10A	0.4166	−0.1657	0.8668	0.144*	
H10B	0.4340	−0.1345	0.7568	0.144*	
H10C	0.3517	−0.1148	0.8298	0.144*	
C11	0.61067 (15)	0.15818 (10)	0.76316 (19)	0.0383 (6)	
C12	0.66082 (17)	0.11905 (11)	0.8257 (2)	0.0478 (6)	
H12	0.6632	0.0797	0.8072	0.057*	
C13	0.70698 (17)	0.13906 (13)	0.9156 (2)	0.0538 (7)	
H13	0.7404	0.1128	0.9571	0.065*	
C14	0.70444 (18)	0.19696 (13)	0.9449 (2)	0.0576 (8)	
H14	0.7357	0.2099	1.0054	0.069*	
C15	0.65481 (18)	0.23514 (13)	0.8829 (2)	0.0577 (8)	
H15	0.6524	0.2744	0.9021	0.069*	
C16	0.60827 (16)	0.21655 (11)	0.7924 (2)	0.0479 (7)	
H16	0.5753	0.2432	0.7512	0.058*	
C17	0.43453 (15)	0.20319 (10)	0.4501 (2)	0.0399 (6)	
C18	0.39468 (17)	0.18210 (12)	0.3574 (2)	0.0505 (7)	
H18	0.3970	0.1423	0.3424	0.061*	
C19	0.35137 (19)	0.21958 (14)	0.2866 (2)	0.0623 (8)	
H19	0.3252	0.2051	0.2239	0.075*	
C20	0.3471 (2)	0.27838 (14)	0.3090 (3)	0.0665 (9)	
H20	0.3182	0.3037	0.2614	0.080*	
C21	0.38543 (19)	0.29943 (13)	0.4014 (3)	0.0646 (8)	
H21	0.3823	0.3392	0.4165	0.077*	
C22	0.42870 (18)	0.26235 (11)	0.4724 (2)	0.0527 (7)	
H22	0.4540	0.2771	0.5354	0.063*	
N1	0.52713 (13)	0.18375 (8)	0.60593 (16)	0.0414 (5)	
N2	0.56296 (14)	0.14038 (8)	0.67014 (16)	0.0444 (5)	
N3	0.63253 (12)	0.04874 (8)	0.61271 (16)	0.0396 (5)	
N4	0.61620 (13)	−0.03289 (9)	0.73112 (17)	0.0430 (5)	
N5	0.47290 (14)	−0.02945 (9)	0.82976 (18)	0.0523 (6)	
N6	0.39730 (15)	0.00565 (10)	0.8391 (2)	0.0618 (7)	
S1	0.47272 (5)	0.08793 (3)	0.51716 (6)	0.0486 (2)	

### Atomic displacement parameters (Å^2^)

**Table:**

	*U*^11^	*U*^22^	*U*^33^	*U*^12^	*U*^13^	*U*^23^
C1	0.0362 (12)	0.0355 (13)	0.0431 (14)	0.0013 (10)	−0.0001 (11)	0.0032 (11)
C2	0.0380 (12)	0.0283 (12)	0.0425 (14)	0.0008 (10)	−0.0069 (10)	−0.0008 (10)
C3	0.0362 (12)	0.0305 (12)	0.0453 (15)	0.0006 (10)	−0.0020 (10)	−0.0003 (11)
C4	0.0440 (14)	0.0471 (16)	0.0621 (19)	0.0096 (12)	0.0070 (12)	0.0006 (14)
C5	0.0365 (12)	0.0331 (13)	0.0435 (14)	−0.0019 (10)	−0.0020 (10)	0.0033 (11)
C6	0.0332 (12)	0.0411 (14)	0.0496 (15)	0.0055 (11)	−0.0048 (11)	−0.0022 (12)
C7	0.0491 (15)	0.0602 (18)	0.0568 (19)	−0.0083 (13)	0.0078 (12)	0.0152 (15)
C8	0.127 (3)	0.107 (3)	0.073 (3)	−0.015 (2)	0.048 (2)	−0.004 (2)
C9	0.070 (2)	0.066 (2)	0.082 (2)	0.0031 (17)	0.0156 (17)	0.0367 (18)
C10	0.118 (3)	0.054 (2)	0.117 (3)	−0.008 (2)	0.038 (3)	0.014 (2)
C11	0.0373 (12)	0.0403 (14)	0.0372 (14)	−0.0053 (10)	0.0004 (10)	−0.0034 (11)
C12	0.0557 (15)	0.0409 (15)	0.0469 (16)	−0.0021 (13)	−0.0091 (12)	0.0008 (12)
C13	0.0506 (15)	0.068 (2)	0.0425 (16)	−0.0036 (14)	−0.0098 (12)	0.0037 (14)
C14	0.0461 (15)	0.075 (2)	0.0516 (18)	−0.0088 (15)	−0.0046 (12)	−0.0178 (15)
C15	0.0515 (15)	0.0553 (18)	0.066 (2)	−0.0024 (14)	−0.0024 (14)	−0.0238 (15)
C16	0.0451 (14)	0.0396 (15)	0.0591 (18)	0.0003 (11)	−0.0071 (12)	−0.0098 (13)
C17	0.0360 (12)	0.0388 (14)	0.0449 (15)	0.0055 (11)	0.0022 (10)	0.0085 (11)
C18	0.0537 (15)	0.0487 (16)	0.0491 (17)	0.0087 (13)	−0.0019 (13)	0.0070 (13)
C19	0.0596 (17)	0.074 (2)	0.0532 (19)	0.0092 (15)	−0.0122 (14)	0.0101 (16)
C20	0.0640 (18)	0.061 (2)	0.074 (2)	0.0148 (16)	−0.0108 (16)	0.0262 (17)
C21	0.0640 (18)	0.0450 (17)	0.085 (2)	0.0131 (14)	−0.0074 (17)	0.0128 (16)
C22	0.0547 (15)	0.0406 (16)	0.0627 (19)	0.0054 (13)	−0.0086 (13)	0.0080 (13)
N1	0.0478 (11)	0.0308 (11)	0.0457 (12)	0.0023 (9)	−0.0042 (10)	0.0065 (9)
N2	0.0595 (13)	0.0278 (11)	0.0461 (13)	−0.0011 (9)	−0.0168 (10)	0.0046 (9)
N3	0.0373 (10)	0.0369 (11)	0.0446 (12)	−0.0005 (9)	0.0006 (8)	0.0041 (9)
N4	0.0377 (11)	0.0395 (12)	0.0519 (14)	0.0061 (9)	−0.0012 (9)	0.0091 (10)
N5	0.0481 (12)	0.0468 (13)	0.0619 (15)	0.0026 (10)	0.0098 (11)	0.0169 (11)
N6	0.0516 (13)	0.0624 (16)	0.0714 (17)	0.0052 (12)	0.0180 (12)	0.0111 (13)
S1	0.0567 (4)	0.0362 (4)	0.0531 (4)	−0.0001 (3)	−0.0217 (3)	0.0025 (3)

### Geometric parameters (Å, º)

**Table:**

C1—N1	1.281 (3)	C10—H10B	0.9600
C1—C17	1.469 (3)	C10—H10C	0.9600
C1—S1	1.749 (2)	C11—C16	1.386 (3)
C2—N3	1.466 (3)	C11—C12	1.393 (3)
C2—C3	1.467 (3)	C11—N2	1.411 (3)
C2—N2	1.487 (3)	C12—C13	1.383 (3)
C2—S1	1.867 (2)	C12—H12	0.9300
C3—C5	1.370 (3)	C13—C14	1.376 (4)
C3—C4	1.401 (3)	C13—H13	0.9300
C4—N6	1.323 (3)	C14—C15	1.371 (4)
C4—H4	0.9300	C14—H14	0.9300
C5—N5	1.352 (3)	C15—C16	1.381 (4)
C5—N4	1.372 (3)	C15—H15	0.9300
C6—N4	1.290 (3)	C16—H16	0.9300
C6—N3	1.361 (3)	C17—C18	1.379 (4)
C6—H6	0.9300	C17—C22	1.386 (3)
C7—N3	1.478 (3)	C18—C19	1.382 (4)
C7—C8	1.482 (4)	C18—H18	0.9300
C7—H7A	0.9700	C19—C20	1.377 (4)
C7—H7B	0.9700	C19—H19	0.9300
C8—H8A	0.9600	C20—C21	1.368 (4)
C8—H8B	0.9600	C20—H20	0.9300
C8—H8C	0.9600	C21—C22	1.378 (4)
C9—N5	1.463 (3)	C21—H21	0.9300
C9—C10	1.486 (5)	C22—H22	0.9300
C9—H9A	0.9700	N1—N2	1.377 (3)
C9—H9B	0.9700	N5—N6	1.364 (3)
C10—H10A	0.9600		
			
N1—C1—C17	121.6 (2)	C12—C11—N2	122.1 (2)
N1—C1—S1	115.96 (18)	C13—C12—C11	119.6 (2)
C17—C1—S1	122.39 (18)	C13—C12—H12	120.2
N3—C2—C3	108.05 (18)	C11—C12—H12	120.2
N3—C2—N2	111.20 (18)	C14—C13—C12	121.4 (3)
C3—C2—N2	114.5 (2)	C14—C13—H13	119.3
N3—C2—S1	111.02 (16)	C12—C13—H13	119.3
C3—C2—S1	110.59 (15)	C15—C14—C13	118.6 (3)
N2—C2—S1	101.41 (14)	C15—C14—H14	120.7
C5—C3—C4	104.8 (2)	C13—C14—H14	120.7
C5—C3—C2	121.5 (2)	C14—C15—C16	121.3 (3)
C4—C3—C2	133.8 (2)	C14—C15—H15	119.3
N6—C4—C3	111.7 (2)	C16—C15—H15	119.3
N6—C4—H4	124.2	C15—C16—C11	120.0 (3)
C3—C4—H4	124.2	C15—C16—H16	120.0
N5—C5—C3	107.4 (2)	C11—C16—H16	120.0
N5—C5—N4	124.9 (2)	C18—C17—C22	119.0 (2)
C3—C5—N4	127.7 (2)	C18—C17—C1	121.3 (2)
N4—C6—N3	129.0 (2)	C22—C17—C1	119.6 (2)
N4—C6—H6	115.5	C17—C18—C19	120.6 (3)
N3—C6—H6	115.5	C17—C18—H18	119.7
N3—C7—C8	114.0 (2)	C19—C18—H18	119.7
N3—C7—H7A	108.8	C20—C19—C18	119.9 (3)
C8—C7—H7A	108.8	C20—C19—H19	120.1
N3—C7—H7B	108.8	C18—C19—H19	120.1
C8—C7—H7B	108.8	C21—C20—C19	119.8 (3)
H7A—C7—H7B	107.6	C21—C20—H20	120.1
C7—C8—H8A	109.5	C19—C20—H20	120.1
C7—C8—H8B	109.5	C20—C21—C22	120.6 (3)
H8A—C8—H8B	109.5	C20—C21—H21	119.7
C7—C8—H8C	109.5	C22—C21—H21	119.7
H8A—C8—H8C	109.5	C21—C22—C17	120.1 (3)
H8B—C8—H8C	109.5	C21—C22—H22	120.0
N5—C9—C10	111.5 (3)	C17—C22—H22	120.0
N5—C9—H9A	109.3	C1—N1—N2	113.15 (19)
C10—C9—H9A	109.3	N1—N2—C11	117.04 (18)
N5—C9—H9B	109.3	N1—N2—C2	118.15 (18)
C10—C9—H9B	109.3	C11—N2—C2	124.77 (19)
H9A—C9—H9B	108.0	C6—N3—C2	122.90 (19)
C9—C10—H10A	109.5	C6—N3—C7	118.6 (2)
C9—C10—H10B	109.5	C2—N3—C7	118.34 (19)
H10A—C10—H10B	109.5	C6—N4—C5	110.84 (19)
C9—C10—H10C	109.5	C5—N5—N6	111.18 (19)
H10A—C10—H10C	109.5	C5—N5—C9	128.3 (2)
H10B—C10—H10C	109.5	N6—N5—C9	120.0 (2)
C16—C11—C12	119.1 (2)	C4—N6—N5	105.0 (2)
C16—C11—N2	118.9 (2)	C1—S1—C2	91.28 (10)

### Hydrogen-bond geometry (Å, º)

Cg1 and Cg2 are the ring centroids of the C17–C22 and C11–C16 rings, respectively.

**Table:**

*D*—H···*A*	*D*—H	H···*A*	*D*···*A*	*D*—H···*A*
C14—H14···*Cg*1^i^	0.93	2.75	3.615 (3)	155
C20—H20···*Cg*2^ii^	0.93	2.77	3.564 (4)	144

Symmetry codes: (i) *x*−1/2, *y*, −*z*+1/2; (ii) *x*−1/2, −*y*+1/2, −*z*+1.
