# Supplementary figures and images for: Crystal structure of 1,5-diethyl-3′,5′-di­phenyl-1,5-di­hydro-3′H-spiro­[pyra­zolo[3,4-d]pyrimidine-4,2′-[1,3,4]thia­diazole]
Source: Acta Crystallogr E Crystallogr Commun. 2015 Sep 26;71(Pt 10):o769–70. doi: 10.1107/S2056989015017405 (PMC4647392; doi:10.1107/S2056989015017405)

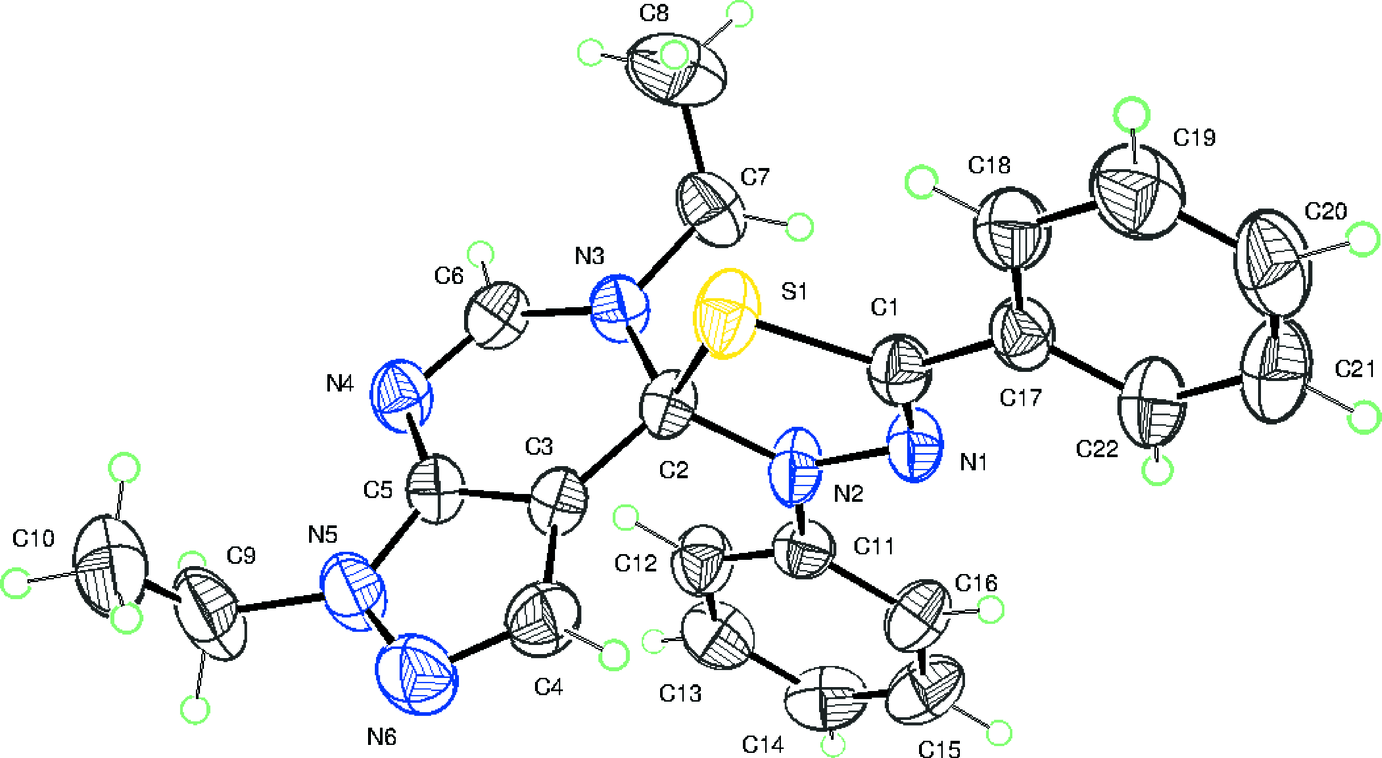

Supplement: Supplementary file 4 [file e-71-0o769-fig1.tif]
